# Supplementary material for: Appendiceal Collision Tumors: An Institutional Case Series and Systematic Review of the Histologic Spectrum, Clinical Outcomes, and Management Strategies
Source: Diagnostics (Basel). 2025 Dec 30;16(1):114. doi: 10.3390/diagnostics16010114 (PMC12785655; doi:10.3390/diagnostics16010114)
Supplement: Supplementary file 1 [file diagnostics-16-00114-s001.zip › diagnostics-4061081-supplementary.pdf]

Supplementary Table S1. Summary of published appendiceal collision tumors, including demographics, component-specific staging, treatment, and follow-up.

No	Reference	Age	Sex	Presentation symptoms	Tumor Components & Staging	Treatment	Follow-up (month)
**1**	Durowoju et al 2025 [15]	mean age 52 years (range 18-77)	F;6, M;9	NS	NEN, G1; pT1 – AC; pT4 (n:3), NEN, G1; pT1 – LAMN; pTis (n:3), NEN, G1; pT2 – LAMN; pTis (n:2), NEN, G1; pT3 – LAMN; pTis (n:3), NEN, G1; pT4 – LAMN pT4 (n:1), NEN, G2; pT3 – LAMN; pTis (n:1), NEN, G2; pT3 – LAMN pT3 (n:1), NEN, G1; pT3 – HAMN; pT4 (n:1)	Simple appendectomy (n: 10), RHC (n:3), RHC (n:3), Cecectomy (n:1), Pelvic exenteration, RHC, proctectomy, partial cystectomy (n:1)	Median follow-up of 11 months (range 1 to 36), the one patient with mucinous AC DCD.
**2**	Hirose et al 2025 [16]	46	F	Incidental	LMAN; pTis - GC AC; pT3	ileocecal resection with regional lymph node dissection	NS
**3**	H. AlAwfi et al. 2024 [17]	57	M	2-month history of abdominal pain	NEN, G1; pT1 - LMAN; pTis	RHC	NS
**4**	Viel et al. 2023 [7]	49, 59	F, F	Incidental	NEN, G1; pT2 - LMAN; pTis, NEN, G1; pT1 - LMAN; pTis	Simple appendectomy, both	Alive (12), both
**5**	Morillo et al. 2023 [18]	20	F	Acute right iliac fossa pain	NEN, G1; pT3 - HAMN; pTis	Simple appendectomy	NS
**6**	Gupta et al. 2023 [19]	64	M	Abdominal pain	NEN, G1; pT2 - LMAN; pT4	RHC and HIPEC	DCD (30)
**7**	Rahman et al. 2022 [20]	65	F	2-week history of abdominal pain	NEN, G1, pT1 - LAMN; pT3 - AC; pT2	RHC	Alive (6)
**8**	Syrine Moussa et al. 2022 [21]	75	M	Acute abdominal pain	NEN, G1; NS - LAMN; Tis	Total Colectomy (Sigmoid colon AC)	NS
**9**	Oka et al 2022 [22]	50	F	Incidental appendix enlargement on CT	AC; pTis - HAMN; pTis	Simple appendectomy	NS
**10**	Melendez et al. 2022 [23]	82, 41	F, M	Acute right iliac fossa pain	GC AC; pT3 - LAMN; pT4, NEN, G1; pT1 - LAMN; pT4	RHC + CRS + HIPEC, Simple appendectomy	NS, Alive (6)
**11**	Ekinci et al. 2021 [24]	60	M	2-month history of non-specific abdominal discomfort	NEN, G1; NS - LAMN; NS	Simple appendectomy	Alive (6)
**12**	Ruiz et al. 2021 [25]	54	M	Abdominal pain, nausea, and vomiting	Mucinous AC; pT2 - NEN, G1; pT3	Appendectomy and cecum resection	NS
**13**	Villa et al. 2021 [10]	31	F	Abdominal pain	NEN, G1; pT3 - LAMN; Tis	RHC	Alive (12)
**14**	Cafaro et al. 2020 [26]	35	F	Appendicitis-like symptoms	NEN, G2; pT3 - LAMN; pTis	Simple appendectomy	Alive (15)
**15**	Carboni et al. 2020 [27]	54	F	A pelvic cystic lesion incidentally detected on ultrasonography	NEN, G1 18 mm pT3 - LAMN Tis - Goblet Cell AC; 8mm (NS)	Simple appendectomy	Alive (9)
**16**	Sholi et al. 2019 [28]	23	F	Right lower quadrant pain	NEN, G2; pT4, N1 - LAMN; pT4	RHC	Alive (24)
**17**	Chinen et al 2019 [29]	75	M	Right lower quadrant pain	Goblet cell AC; pT4 - LAMN; NS	CRS + CHT	DCD (7)
**18**	Hajjar et al.2019 [30]	50	M	Abdomino-pelvic pain	NEN, G2; T3 - LAMN; T4, M1	RHC+ CRS + HIPEC	Alive (20)

**19**	Yeh et al. 2018 [31]	66	F	Persistent right lower quadrant pain	AC, Tis - NEN, NS	Simple appendectomy	NS
**20**	Sato et al 2018 [32]	78	M	Not had specific symptoms	Goblet cell AC; pT3 - LMAN Tis	NS	NS
**21**	R. Das et al 2017 [33]	67	F	Right lower quadrant pain	NEN, G1; NS - LAMN; Tis	RHC	NS
**22**	Sugarbaker et al. 2016 [34]	39, 32	F, F	Right lower quadrant pain, Incidental	NEN, G1; pT1 - LAMN; pT3, N0, M1(n:1) NEN, G2; T2, N1 - LAMN; T3, M1	RHC+ CRS+HIPEC, Appendectomy + CRS + HIPEC	Alive (60), (12)
**23**	Tan et al. 2015 [35]	52	M	Persistently elevated serum carcinoembryonic antigen	NEN, G1; NS - LAMN; Tis	Simple appendectomy	NS
**24**	Baena-del-Valle et al. 2015 [36]	49, 45	F, F	Observed in abdominal CT for hernia evaluation - Progressive abdominal distention.	NEN, G1; NS - LAMN; T4; both	Appendectomy + CRS + HIPEC	NS, DCD (24)
**25**	Ng et al. 2014 [37]	58, 47	F, F	NS	LAMN and Goblet cell AC, both NS	NS	NS
**26**	Dellaportas et al. 2014 [38]	57	F	Incidentally palpating a mass on right iliac fossa	NEN G1; pT4 - LAMN; Tis	RHC	Alive (12)
**27**	Singh et al. 2010 [39]	52	M	Pain in the right lower quadrant.	Mucinous AC; pT3, N1, M1 - NEN, G1; pT3	CHT	DCD (14)
**28**	Chetty et al. 2010 [40]	47	M	Appendicitis-like symptoms	Goblet Cell AC - NEN, G1; pT1	Simple appendectomy	Alive (6)
		52	F	Pelvic mass	Goblet Cell AC - NEN, G1; pT4	RHC and pelvic extension	DCD (6)
		53	M	Appendicitis-like symptoms	Goblet Cell AC - NEN, G1; pT3	RHC	NS
		52, 63	M	Appendicitis-like symptoms	Goblet Cell AC - NEN, G1; pT1, both	RHC, both	Alive (54), (9)
**29**	Alsaad Et al. 2009 [41]	46	F	Right lower quadrant pain	LAMN; pTis - GC AC; pT3, N0	RHC	NS
**30**	Rossi et al. 2004 [42]	35	F	Right lower quadrant pain	Mucinous AC; pT3, N1- NEC; NS	RHC	Alive (65)
**31**	Al-Talib et al. 1995 [43]	54, 64	F	Appendicitis-like symptoms / four-month history of a dull ache in the right iliac fossa	LAMN; pT4- Goblet Cell AC; NS (both)	NS	NS
**32**	Carr et al. 1995 [6]	Mean; 49 (22 to 92)	7F, 6M	Appendicitis-like symptoms; (4), Chronic lower abdominal pain; (3), abdomino-pelvic mass (3), Mucin in hernia sac; (1), Small bowel obstruction; (1), Incidental; (1)	Goblet cell AC (3) and NEN (10), mucinous cystadenoma (4), Mucinous tumor uncertain malign potential (3) and Mucinous AC (6)	NS	NS
**33**	Sjövall et al. 1985 [44]	73	M	Right lower quadrant pain	AC, NS- NEN, NS	RHC	Alive (3)

Supplementary Table S1 summarizes published appendiceal collision tumors, including demographics, clinical presentation, component-specific pathologic staging, treatment, and follow-up. Pathologic stage is reported separately for each component as described in the original reports. Follow-up duration and disease status are shown when available; otherwise, entries are coded as not specified (NS). Abbreviations: AC, adenocarcinoma; DCD, dead of disease; HAMN, high-grade appendiceal mucinous neoplasm; LAMN, low-grade appendiceal mucinous neoplasm; NEN, neuroendocrine neoplasm.
